# The Microscopic Mechanism of High Temperature Resistant Core-Shell Nano-Blocking Agent: Molecular Dynamics Simulations

**DOI:** 10.3390/polym17141969

**Published:** 2025-07-17

**Authors:** Zhenghong Du, Jiaqi Xv, Jintang Wang, Juyuan Zhang, Ke Zhao, Qi Wang, Qian Zheng, Jianlong Wang, Jian Li, Bo Liao

**Affiliations:** 1 Drilling Engineering Research Institute, Sinopec Southwest Petroleum Engineering Co., Ltd., Deyang 618000, China; duzh.osxn@sinopec.com; 2 School of Petroleum Engineering, China University of Petroleum (East China), Qingdao 266580, China; s23020130@s.upc.edu.cn (J.X.); wangjintang@upc.edu.cn (J.W.); zhangjy0423@163.com (J.Z.); b24020053@s.upc.edu.cn (K.Z.); wang-qi2025@163.com (Q.W.); s24020174@s.upc.edu.cn (Q.Z.); cuplijian@sina.com (J.L.); 3 CNPC Engineering Technology R&D Co., Ltd., Beijing 102206, China; wjldr@cnpc.com.cn

**Keywords:** drilling fluid, plugging agent, molecular simulation, shale reservoir

## Abstract

China has abundant shale oil and gas resources, which have become a critical pillar for future energy substitution. However, due to the highly heterogeneous nature and complex pore structures of shale reservoirs, traditional plugging agents face significant limitations in enhancing plugging efficiency and adapting to extreme wellbore environments. In response to the technical demands of nanoparticle-based plugging in shale reservoirs, this study systematically investigated the microscopic interaction mechanisms of nano-plugging agent shell polymers (Ployk) with various reservoir minerals under different temperature and salinity conditions using molecular simulation methods. Key parameters, including interfacial interaction energy, mean square displacement, and system density distribution, were calculated to thoroughly analyze the effects of temperature and salinity variations on adsorption stability and structural evolution. The results indicate that nano-plugging agent shell polymers exhibit pronounced mineral selectivity in their adsorption behavior, with particularly strong adsorption performance on SiO_2_ surfaces. Both elevated temperature and increased salinity were found to reduce the interaction strength between the shell polymers and mineral surfaces and significantly alter the spatial distribution and structural ordering of water molecules near the interface. These findings not only elucidate the fundamental interfacial mechanisms of nano-plugging agents in shale reservoirs but also provide theoretical guidance for the precise design of advanced nano-plugging agent materials, laying a scientific foundation for improving the engineering application performance of shale oil and gas wellbore-plugging technologies.

## 1. Introduction

China possesses abundant shale oil and gas resources with broad development prospects [1,2,3,4,5,6]. However, shale gas reservoirs are typically buried at depths exceeding 3500 m, with formation pressure coefficients ranging from 1.35 to 2.03. The harsh conditions—high temperature, high pressure, and high salinity—impose stringent requirements on the performance of drilling fluid additives [7,8,9,10,11,12]. During drilling, shale formations exhibit micro- to nano-scale pores (diameter < 100 nm) and natural fracture networks, leading to frequent wellbore instability and drilling fluid loss [13,14,15,16,17,18]. Conventional plugging agents (e.g., asphalt-based materials and polymer microspheres), with particle sizes typically in the micrometer range, struggle to effectively seal nanoscale fractures and degrade under high-pressure, high-temperature (HPHT) conditions [19,20,21]. Meanwhile, conventional water-based drilling fluids suffer from insufficient inhibition and poor lubricity. Consequently, the development of novel plugging agents that combine nanoscale sealing capability with high-temperature resistance has become critical for improving the efficiency of shale gas drilling.

Nanoparticle-based plugging agents have achieved performance breakthroughs through inorganic–organic composite design [22,23], and a series of intelligent-response-enabled nano-capsule plugging agents have been developed [24,25]. However, the underlying mechanisms of their plugging action remain unclear, and the influence of shell materials on plugging performance has not been fully elucidated. In recent years, molecular simulation has been widely applied in petroleum chemistry research [26,27,28,29,30,31]. For instance, Dong et al. [32] employed molecular dynamics (MD) simulations to investigate the effect of salt concentration on the dispersion stability of nano-emulsion-based plugging materials. By analyzing the responsiveness of monomers to salt concentration, their study revealed the mechanism by which salt ions weaken the dispersion stability of nanoparticle-based plugging agents. Thus, molecular simulation can be used to calculate intermolecular interactions within drilling fluids, as well as interactions between drilling fluid components and formation rocks [33,34]. This approach enables the elucidation of plugging agent mechanisms under varying environmental conditions and facilitates the prediction of performance variations in different scenarios.

In this study, molecular dynamics (MD) simulations were employed to investigate the effects of temperature, salinity, and mineral type on the interaction energies between nano-plugging agent shell polymer (Ployk) molecules and mineral surfaces. By calculating key parameters—including interaction energy, mean squared displacement (MSD), radial distribution functions (RDF), and density distributions—the variation patterns of these properties were systematically analyzed. The findings provide valuable insights for the development of novel nanoparticle-based plugging agents.

## 2. Methods

**Figure 1:**
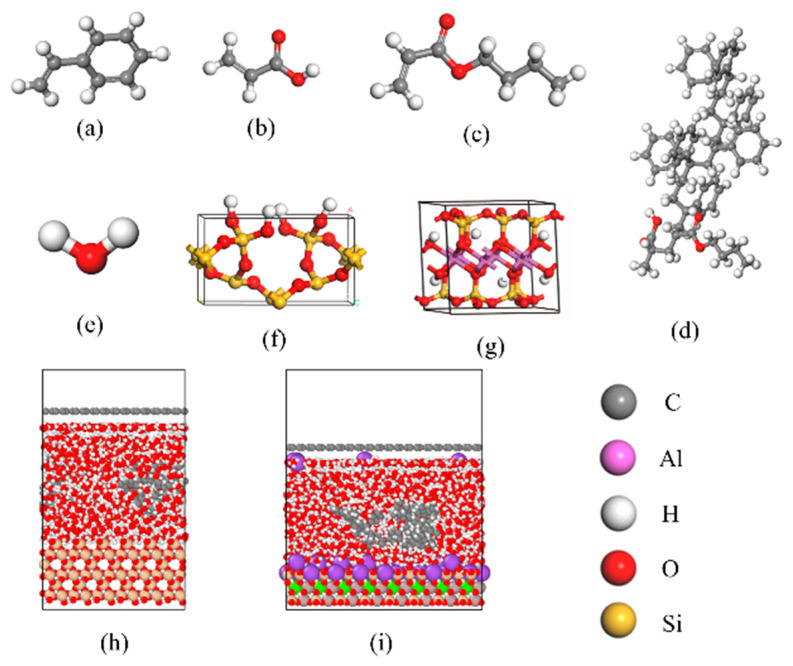
Molecular models: (**a**) styrene, (**b**) acrylic acid, (**c**) butyl acrylate, (**d**) Ployk polymer, (**e**) water molecule, (**f**) SiO_2_, (**g**) montmorillonite, (**h**) SiO_2_ adsorption model, and (**i**) montmorillonite adsorption model.

Molecular dynamics (MD) simulations were performed using the synthesized high-temperature-resistant nano-capsule plugging agent [35]. Ployk has a unique core-shell-like structure, which not only provides rigidity to ensure mechanical plugging capability but also possesses flexibility to adapt to the morphology of shale nano-pores and fractures. Based on the monomer feeding molar ratio during experimental synthesis, the molecular composition in the MD model was set to a styrene (St): butyl acrylate (BA): acrylic acid (AA) ratio of 10:2:1. Considering the nanoscale dimensions of the plugging agent and its practical interaction mechanisms, the adsorption process between the nanocapping agent and formation minerals was simplified to the interaction between the Ployk polymer shell and the mineral surfaces. The mineral framework models included SiO_2_ and montmorillonite (Figure 1), and the molecular structure of Ployk is also shown in Figure 1.

The molecular simulation system in this study consisted of three Ployk molecules and 700 water molecules. When studying the influence of different salinities, the molecular simulation system consists of Ployk molecules, Na^+^, Cl^−^, and water molecules in a certain proportion. The simulation box dimensions were set to 30 Å × 30 Å × 23 Å along the x, y, and z directions, respectively. For the mineral phase, SiO_2_ and montmorillonite unit cells were replicated in a 6 × 6 × 2 array along the x, y, and z directions. The SiO_2_ unit cell was modified by adding hydrogen atoms to the surface oxygen atoms of the silicon dioxide unit cell to create a hydrophilic SiO_2_ surface.

The simulations in this study were performed using LAMMPS software (23 Jun 2022—Update 4) [36]. The accurate selection of molecular force fields is critical for precisely calculating atomic interactions. Based on previous studies, the ClayFF force field [37] was employed to describe montmorillonite and SiO_2_, with all mineral frameworks maintained as rigid bodies during the simulations. For water molecules, the SPC/E model [38] was adopted, and bond lengths and angles were constrained using the SHAKE algorithm. The Ployk molecules were described using the OPLS-AA force field [39]. A time step of 1 fs was used, with a cutoff radius of 1.25 nm, and long-range interactions were calculated using the PPPM algorithm. The solution system was simulated under the NVT ensemble, with temperature control achieved via the Nosé–Hoover thermostat [40].

## 3. Result and Discussion

### 3.1. Interaction Studies Between Ployk Molecules and Different Minerals

**Figure 2:**
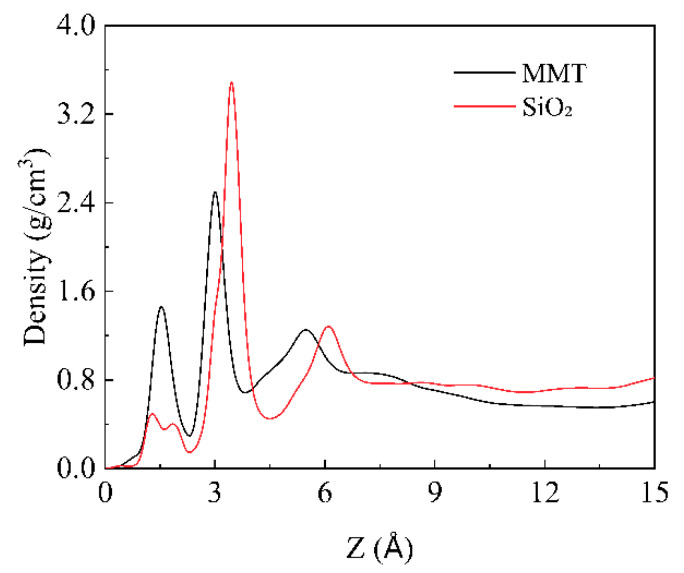
Density distribution of water molecules on different mineral surfaces.

Prior to investigating the interaction energies between Ployk molecules and montmorillonite (MMT) or SiO_2_, the distribution characteristics of water molecules on the surfaces of these two clay minerals were analyzed (Figure 2). The results reveal that due to the relatively large distance between surface hydroxyl groups on SiO_2_, water molecules exhibit a smaller first adsorption peak at 1.29 Å, with an adsorption layer thickness of 3.8 Å. In contrast, the presence of surface Na^+^ ions on MMT leads to a distinct ion hydration peak at 1.54 Å, resulting in a thicker adsorption layer (4.4 Å). These findings demonstrate that MMT supports a more extensive water adsorption layer compared to SiO_2_.

The interaction energy between Ployk polymer molecules and different mineral surfaces is the sum of hydrogen bonding energy, van der Waals energy, and electrostatic energy. The calculation follows Equation (1):

(1) $$E_{int}=E_{AB}-\left(E_{A}+E_{B}\right)$$

where *E_AB_* represents the total energy of the *AB* system, while *E_A_* and *E_B_* denote the energies of components *A* and *B*, respectively.

As derived from Equations (1)–(3), a positive interaction energy indicates repulsive forces between the two components, whereas a negative value signifies attractive interactions. Notably, the magnitude of the negative interaction energy correlates directly with the strength of the attractive interaction—larger absolute values represent stronger binding between the components.

**Figure 3:**
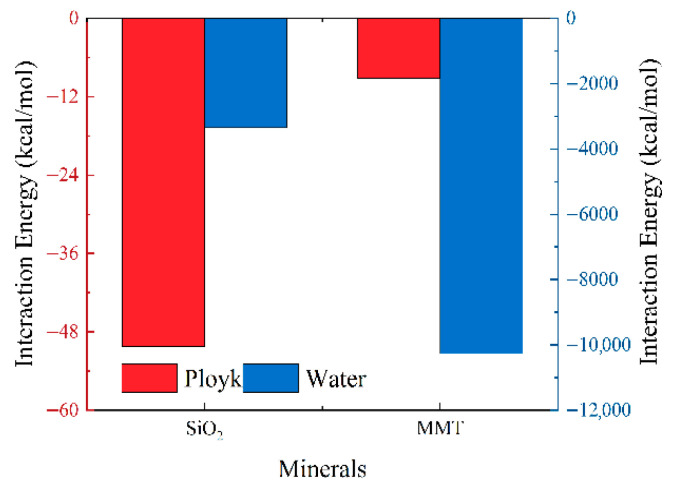
Interaction energies between Ployk molecules/water and montmorillonite (MMT)/SiO_2_ surfaces.

The calculated interaction energies between Ployk polymer molecules, water molecules, and different mineral surfaces are presented in Figure 3. The results show that Ployk exhibits interaction energies of −50.3 kcal/mol with SiO_2_ and −9.2 kcal/mol with MMT. This demonstrates that Ployk forms stronger interactions with SiO_2_ than with MMT, which correlates well with the adsorption distribution patterns shown in Figure 2. Similarly, water demonstrates significantly stronger binding with MMT (−10277.9 kcal/mol) compared to SiO_2_ (−3349.3 kcal/mol). This enhanced interaction with MMT can be attributed to the formation of an electrical double layer due to MMT’s unique surface charge distribution. The comparative analysis reveals that for shale oil and gas reservoirs, Ployk’s stronger adsorption affinity for SiO_2_ surfaces will lead to preferential adsorption on SiO_2_, thereby exhibiting enhanced aggregation and plugging capabilities.

**Figure 4:**
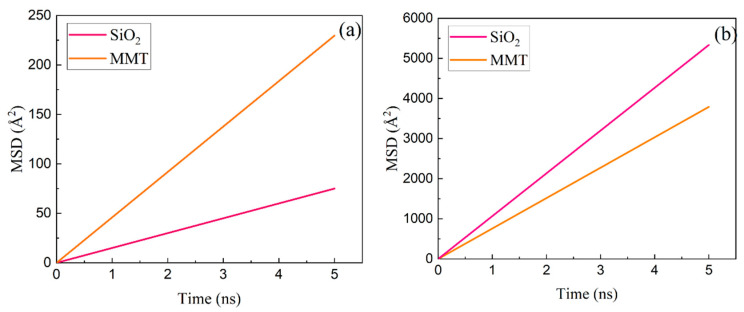
The MSD of (**a**) Ployk polymer molecules and (**b**) water molecules under different mineral surface conditions.

The MSDs of Ployk polymer molecules and water molecules on both mineral surfaces were calculated, as shown in Figure 4. The MSD, representing the statistical average of squared displacements at time t [41], is defined as:

(2) $$MSD=<\frac{1}{N}\sum_{i=1}^{N}{\left[r_{i}\left(t_{0}+t\right)-r_{i}\left(t_{0}\right)\right]}^{2}>$$

where *N* represents the constant number of atoms in the molecular group, r_i_(t_0_ + t) denotes the position of atom i at time t_0_ + t, and r_i_(t_0_) represents the initial position of atom i at time t_0_. This definition of MSD effectively characterizes the relative displacement of atoms within the group from their initial positions, thereby providing a direct measure of the group’s diffusion behavior, expressed as:

(3) $$D=\frac{1}{4}\underset{t\to \infty }{\lim}\frac{\langle |\vec{r}\left(t_{0}+t\right)-{\left.\vec{r}\left(t_{0}\right)\right|}^{2}\rangle }{t}$$

where *D* represents the molecular diffusion coefficient.

**Figure 5:**
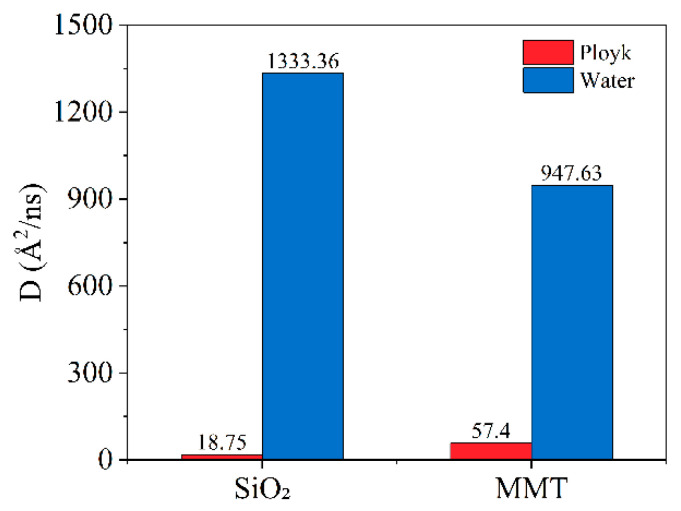
Diffusion coefficients of Ployk polymer molecules and water molecules on different mineral surfaces.

The statistical results of diffusion coefficients for Ployk polymer molecules and water molecules on both mineral surfaces are presented in Figure 5. On SiO_2_ surfaces, Ployk exhibits a diffusion coefficient of 18.75 Å^2^/ns, while this value increases to 57.4 Å^2^/ns on MMT surfaces. Conversely, water demonstrates a significantly higher diffusion coefficient on SiO_2_ (1333.36 Å^2^/ns) that decreases to 947.63 Å^2^/ns on MMT surfaces. This contrasting behavior can be attributed to the electrical double layer formed on MMT surfaces, which restricts water molecule mobility while simultaneously enhancing their adsorption energy through stronger electrostatic interactions. The results demonstrate that Ployk molecules exhibit more restricted mobility on SiO_2_ surfaces compared to MMT. When correlated with the previously discussed interaction energy results, these findings reveal that Ployk molecules form stronger interactions with SiO_2_ surfaces while simultaneously exhibiting reduced mobility. This combination of properties suggests superior plugging performance for Ployk in shale reservoir applications.

### 3.2. Effect of Temperature on the Interaction Between Ployk and SiO_2_

**Figure 6:**
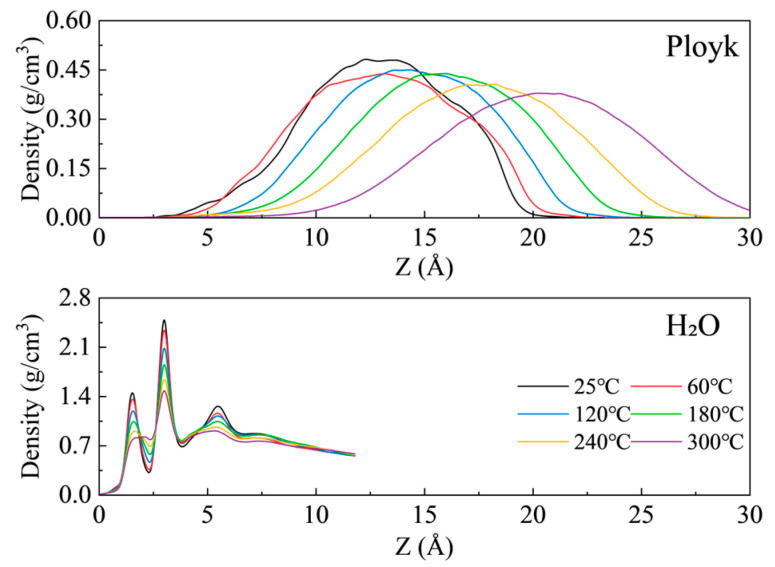
Distance-dependent density distribution profiles of water molecules on SiO_2_ surfaces at varying temperatures.

The aforementioned results demonstrate that Ployk molecules exhibit stronger adsorption affinity for SiO_2_ surfaces. Consequently, elucidating the effects of temperature and pressure conditions on the interactions between Ployk molecules and SiO_2_ is crucial for analyzing Ployk’s plugging performance under extreme HPHT conditions. We first examined the distribution characteristics of Ployk molecules and H_2_O on SiO_2_ surfaces within the temperature range of 25–300 °C. Figure 6 presents the density distribution curves at 140 MPa across different temperatures. The results reveal that water molecules form two distinct adsorption peaks on the SiO_2_ surface, located at 1.57 Å and 3.02 Å from the surface, respectively. With increasing temperature, water molecules exhibit a decreasing trend, manifested as reduced molecular density due to enhanced thermal motion. In contrast, for Ployk molecules, the adsorption peak shifts progressively from 12.27 Å to 20.62 Å with rising temperature, indicating a movement away from the surface. These findings suggest that Ployk’s adsorption capacity needs to be enhanced to maintain its effectiveness under high-temperature conditions.

**Figure 7:**
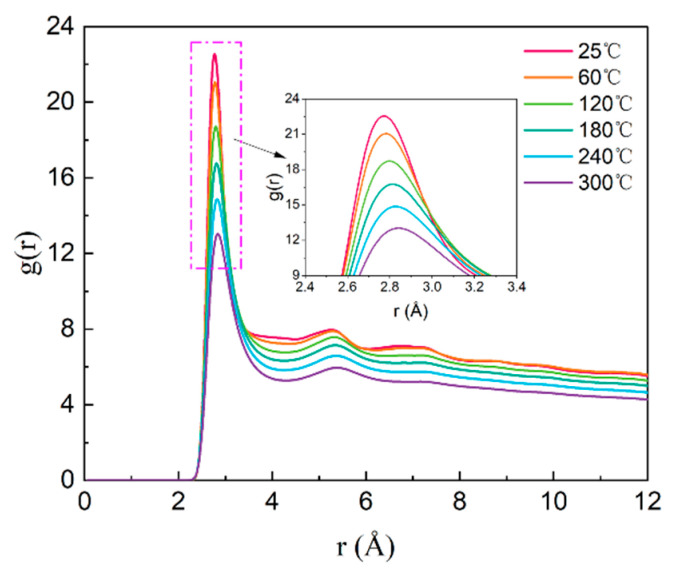
Radial distribution functions (RDF) of O–O pairs in water molecules at varying temperatures.

The radial distribution functions g(r) of O–O pairs in water molecules at various temperatures are presented in Figure 7. Across all tested temperatures, the position of the first peak remains constant at 2.81 Å, while the peak intensity progressively decreases from 23.49 to 13.55 with increasing temperature. This observation indicates increased spatial dispersion of water molecules, which is consistent with the well-established principle that higher temperatures intensify molecular thermal motion.

**Figure 8:**
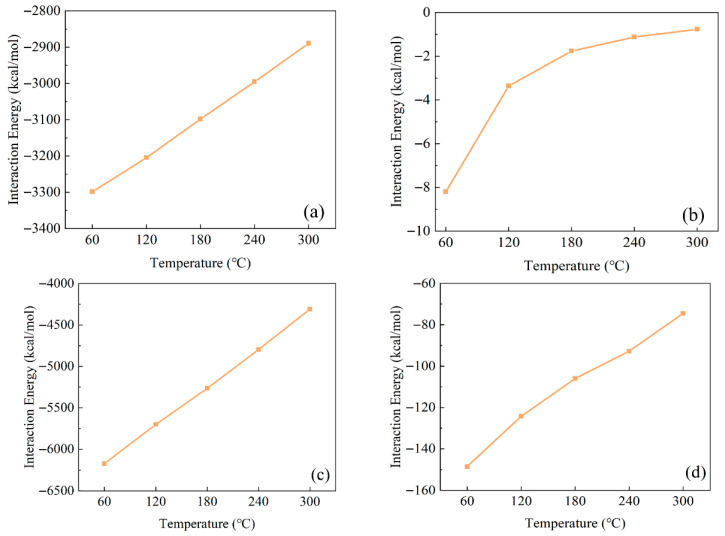
Temperature-dependent interaction energies between different components: (**a**) water–SiO_2_, (**b**) Ployk–SiO_2_, (**c**) water–water, and (**d**) water–Ployk systems.

The calculated interaction energies between Ployk–SiO_2_ systems at varying temperatures are presented in Figure 8. As shown in panel (a), the interaction energy reaches a minimum of −8.204 kcal/mol at 60 °C, gradually increasing to −0.769 kcal/mol at 300 °C. This temperature-dependent trend demonstrates that rising temperatures weaken the interactions between Ployk and SiO_2_ surfaces. Notably, water–SiO_2_ interactions (panel b) exhibit similar temperature dependence. Panels (c) and (d) further reveal that both water–water and water–Ployk interactions within the solution follow the same pattern—interaction energies increase with rising temperature.

**Figure 9:**
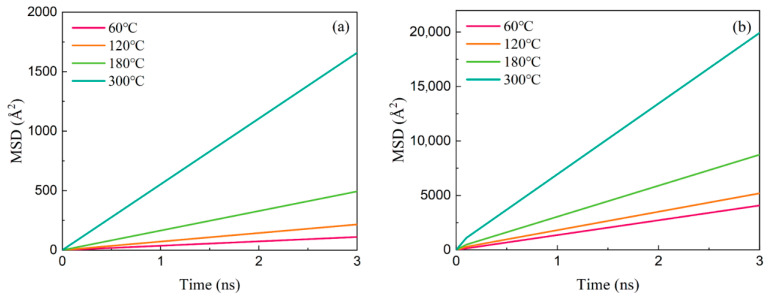
Temperature-dependent MSD: (**a**) Ployk molecules, (**b**) water molecules.

Figure 9 presents the temperature-dependent evolution of MSD for Ployk molecules and water molecules. At 60 °C, Ployk exhibits a MSD of 110.38 Å^2^ at 3 ns, while water molecules demonstrate a significantly higher MSD of 4082.31 Å^2^ under identical conditions. With increasing temperature, thermal motion enhancement leads to progressive increases in MSD values for both systems. By 300 °C, the 3 ns MSD values rise to 1658.24 Å^2^ for Ployk and 19958.17 Å^2^ for water molecules. These findings are complemented by Table 1, which summarizes the corresponding diffusion coefficients. The data reveal systematic increases in diffusivity with temperature, and the diffusion coefficient of Ployk rises from 27.59 Å^2^/ns at 60 °C to 414.55 Å^2^/ns at 300 °C, while water exhibits a more substantial increase from 1020.58 Å^2^/ns to 4989.54 Å^2^/ns over the same temperature range.

### 3.3. Effect of Salinity on the Interaction Between Ployk and SiO_2_

**Figure 10:**
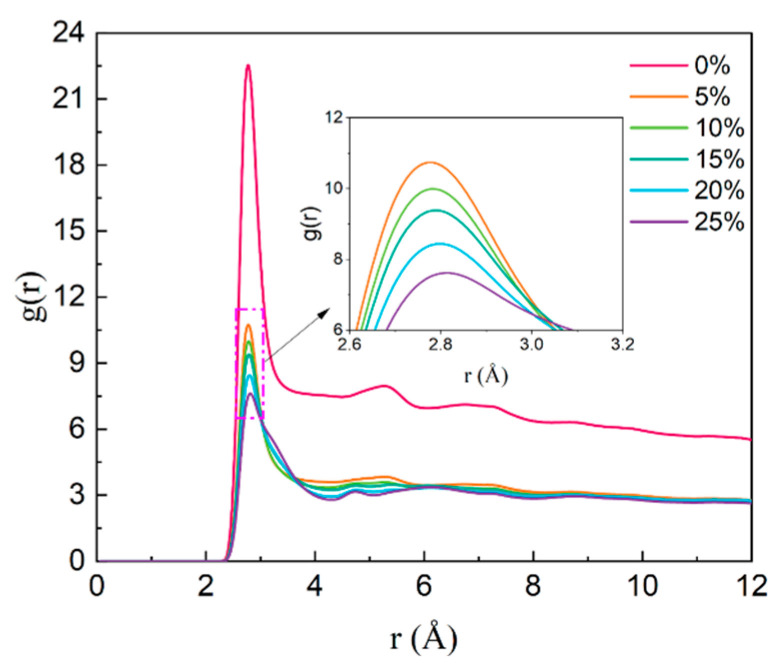
Salinity-dependent radial distribution functions (RDF) of O–O pairs in water molecules.

As shown in Figure 10, the first peak position of water’s radial distribution function g(r) remains constant at 2.8125 Å across all salinity conditions. However, the peak intensity exhibits a notable decrease with increasing salinity, declining from 23.49 at 0% salinity to 8.02 at 25% salinity. The most significant reduction occurs between 0% and 5% salinity (from 23.49 to 11.24), indicating rapid formation of a hydration layer due to low-concentration salt ion intercalation. This alters the arrangement of water molecules. At higher salinity levels (5–25%), the peak intensity continues to decrease (from 11.24 to 8.02), though the rate of change diminishes. This suggests that beyond a certain concentration threshold, additional salt ions have progressively less impact on water molecule rearrangement.

**Figure 11:**
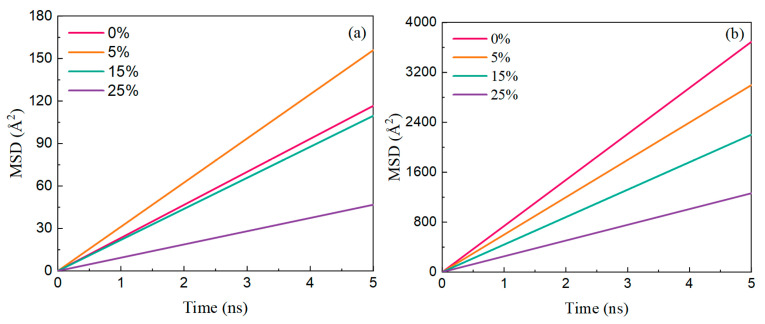
Salinity-dependent MSD of (**a**) Ployk molecules and (**b**) water molecules.

Figure 11 illustrates the salinity-dependent MSD of Ployk molecules and water molecules at 5 ns simulation time, and the diffusion coefficients are shown in Table 2. In the absence of salt (0% NaCl), Ployk molecules exhibit a diffusion coefficient of 29.16 Å^2^/ns, which increases sharply to 38.99 Å^2^/ns upon exposure to 5% NaCl solution, indicating an initial enhancement in molecular mobility due to transient electrostatic interactions with free ions. However, as the salinity continues to increase, the diffusion coefficient of Ployk molecules gradually decreases, reaching 11.69 Å^2^/ns at 25% NaCl concentration, and reflecting the dominant effect of ionic strength that restricts molecular motion, which is primarily attributed to the enhanced interaction between Ployk and ions (Na^+^ and Cl^−^). As salinity rises, the concentration of Na^+^ and Cl^−^ in the system increases, and these ions tend to interact with the polar groups in Ployk’s polymer chain. This ionic interaction reduces the flexibility of Ployk’s molecular chain and increases the “hydration shell” effect around the polymer, creating additional steric hindrance to its movement. Furthermore, higher ion concentrations intensify the competition between ions and Ployk for interactions with water molecules, weakening the solvation of Ployk in the system.

In contrast, the diffusion coefficient of water molecules shows a consistently decreasing trend with rising salinity, from 922.52 Å^2^/ns in pure water to 550.43 Å^2^/ns at 25% NaCl. This pronounced reduction in water mobility can be attributed to the formation of a more structured hydration shell around ions, increased electrostatic shielding effects, and stronger ion–water interactions that collectively constrain the free diffusion of water molecules. The underlying mechanism involves the addition of abundant positive and negative charges from dissolved salts, which significantly amplifies the Coulombic forces in the system, thereby altering the original motion patterns of both Ployk molecules and water molecules through ionic attraction effects. This comprehensive analysis demonstrates the complex and concentration-dependent influence of salinity on molecular dynamics in aqueous solutions.

**Figure 12:**
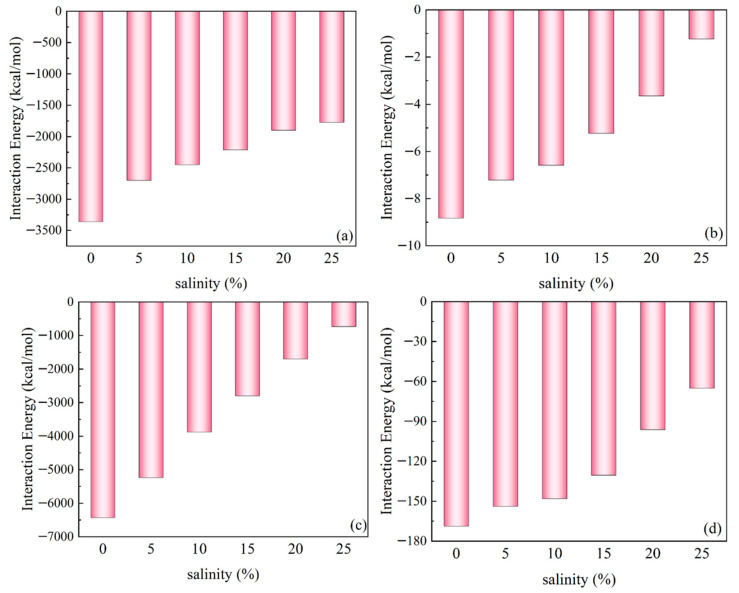
Salinity-dependent interaction energies between different molecular components: (**a**) water–SiO_2_, (**b**) Ployk–SiO_2_, (**c**) water–water, and (**d**) water–Ployk systems.

The interaction energy variations between different molecular systems under varying salinity conditions are systematically presented in Figure 12. For the Ployk-SiO_2_ system (Figure 12a), the interaction energy exhibits a concentration-dependent increase, with a gradual rise from 5% to 15% NaCl followed by a sharp increase at higher concentrations. This suggests an initial stabilization effect at moderate salinities, transitioning to stronger ionic interactions at elevated concentrations. The water–SiO_2_ interactions (Figure 12b) demonstrate a consistent decrease in interaction energy with increasing salinity, indicating progressive weakening of water–SiO_2_ binding. The reduced attraction at higher salt concentrations implies enhanced competition from free ions. For water–water interactions (Figure 12c), the interaction energy increases dramatically from −6431.77 kcal/mol in pure water to −732.93 kcal/mol at 25% NaCl. This substantial reduction in negative interaction energy reflects significant disruption of the hydrogen-bonding network by dissolved salts. The water–Ployk interactions (Figure 12d) show a general increasing trend with salinity, where higher NaCl concentrations systematically weaken the water–Ployk interactions. This behavior parallels the observations for water–SiO_2_ systems, suggesting similar competitive mechanisms between free ions and molecular interactions.

## 4. Conclusions

This study systematically investigated the relationships between mineral types, temperature, and salinity on the interactions between nanoscale sealants and clay minerals using molecular simulation techniques. The key findings are as follows:

1. Ployk exhibits distinct adsorption characteristics on different mineral components. Analysis of interaction energy profiles and density distribution results demonstrates that Ployk shows strong adsorption affinity for reservoir mineral (SiO_2_), while exhibiting weaker adsorption for swelling clay mineral montmorillonite. These results indicate robust interactions between the nanosealant and wellbore walls, which is favorable for sealing micro-fractures and pores.
2. Temperature demonstrates a significant influence on Ployk’s behavior. Elevated temperatures reduce the interaction strength between Ployk and SiO_2_, simultaneously accelerating Ployk’s mobility. This temperature-dependent behavior suggests potential impacts on sealant performance under varying downhole conditions.
3. Salinity shows comparatively minor effects on Ployk’s interactions. While increased salinity slightly weakens the Ployk–SiO_2_ interaction, the variation remains negligible within the 5–15% salinity range. This indicates that the nanosealant possesses considerable salt tolerance, maintaining its fracture-sealing capabilities under moderate salinity conditions.

## Figures and Tables

**Table 1:** Diffusion coefficients under varying temperature conditions.

	Ployk (Å^2^/ps)	Water (Å^2^/ps)
60 °C	27.59	1020.58
120 °C	53.92	1299.84
180 °C	123.32	2188.59
300 °C	414.55	4989.54

**Table 2:** Diffusion coefficients under varying salinity conditions.

	Ployk (Å^2^/ps)	Water (Å^2^/ps)
0%	29.16	922.517
5%	38.99	749.19
15%	27.40	693.96
25%	11.69	550.43

## Data Availability

Data will be made available on request.
